# Conflict and health: a paradigm shift in global health and human rights

**DOI:** 10.1186/1752-1505-1-1

**Published:** 2007-03-01

**Authors:** Sonal Singh, James J Orbinski, Edward J Mills

**Affiliations:** 1 Wake-Forest University Health Sciences, Winston-Salem, North Carolina, USA; 2 University of Toronto, Toronto, Ontario, Canada; 3 Centre for International Health and Human Rights Studies, North York, Ontario, Canada

## 

We are not born equal. The possibility for equality must first be imagined, and then actively created. The 1948 *United Nations Declaration of Human Rights *[1] is one such imagining, and it is far from being fully realized. The day before it was signed in Paris, the *United Nations Convention on the Prevention and Punishment of Genocide *[2] was passed. Its aspirations too, are far from being fully realized. The practice of humanitarianism in war, while having evolved since its formal inception in 1864 [3], today risks being overwhelmed by both military and political agendas. Rapid advances in public health and modern medicine have increased life expectancy in many countries by several decades, though widening inequalities between developed and developing countries and within various national groups continue to exist and in many cases flourish. In each of these domains, however, we are further along, though by how much, and in what direction, is not always known.

War is one of the world's most serious threats to health. The lives of millions around the world are caught between the vicious spiral of violent conflict and poor health. Health professionals around the world have been participating in the emerging discipline of health and human rights [4]. They have attempted to tackle some of these issues through advocacy and participation in global health challenges such as access to medicines for HIV and other neglected diseases [5]. Although intuitively appealing, the legal complexities of health and human rights principles, and poorly formulated evidence in advancing the cause of global health, have reduced human rights arguments to lofty ideals that are widely quoted in academic circles but seldom implemented. The forces of globalization responsible for the spread of some of the advances of the last century have raised discontent among people around the world on several important issues. It is not a coincidence that nations that endure some of the most violent and protracted conflicts also suffer some of the worst health indicators [6]. It is also not a coincidence that, for example, trade rules, increased economic globalization, and a lack of evidence-based interventions have impacted both positively and negatively on the ability of countries to respond to the HIV/AIDS pandemic and other disease conditions [7].

Health professionals, and most current medical journals barring a few exceptions – *The Lancet*, *PLoS Medicine*, *BMC International Health and Human Rights *– have been effectively neutral in the debate on why the health of the majority of the world's population continues to wane while failing to meet its full potential. We believe health professionals have a duty to report on health and human rights among vulnerable populations.

One of the most controversial issues within the humanitarian community is the use of evidence to inform humanitarian responses. Data collection is one of the areas of humanitarianism that has the most to benefit from medicine and epidemiology. For several reasons, including ethical quandaries; philosophical differences; lack of infrastructure; and a lack of funding; the humanitarian field has been reticent to apply methodological principles in data collection and application. However, without the development of an evidence-base for interventions, we cannot be sure if our actions result in more benefit than harm for the vulnerable populations we work with. Existing publications in this discipline are confined to articles published in general medical journals on medical consequences of conflict. These articles sometimes fail to elucidate the multidimensional relationship between health and conflict and evaluate it in an evidence based epidemiological approach. This journal is part of a growing effort to develop accessible evidence for humanitarian responses.

*Conflict and Health *will explore the relationships between health, human rights, humanitarianism and conflict. The journal seeks to explore both the practice and the discipline of health as a right. The practice explores the conditions, limitations and challenges of achieving health, while the discipline seeks to explore how to make the imagined real. It is rooted in a particular imagining of human dignity – a view that sees any one human being as intimately related to all others. It will encourage the development of an evidence base for an emerging discipline. We will engage readers from around the world and stimulate debate in this field by publishing research that emphasizes originality, cross-disciplinarity, and sound methodology. It would include reviews that advance our understanding of this evolving field and invited commentaries from experts around the world in the field of health and conflict. In keeping with the principles of the journal and Editorial Board, *Conflict and Health *is open-access and freely available to readers around the world. In the past, human rights workers, lawyers, health professionals and epidemiologists have chosen to work in isolation within their own fields to the detriment of health and human rights. Our multidisciplinary editorial team includes physicians, nurses, public health specialists, social scientists, lawyers, psychologists, anthropologists, social workers and conflict experts from the northern and southern hemispheres. Open access will promote interdisciplinary research through innovative partnerships between academic and private researchers [8] as well as through their interaction with the human rights community. [8] We encourage authors from all disciplines including health professionals at all levels of training, health researchers, anthropologists and social scientists interested in the interplay of health and conflict to contribute. The journal will serve as an indispensable resource for international non-governmental organisations, donors and policy makers providing accurate and timely information for policy making for the reconstruction of health systems in conflict settings.

We reaffirm that health is a practical daily concern with political, structural, social as well as biophysical dimensions. We encourage participation from around the world, as exemplified in our first set of articles from Chechnya to Uganda to Geneva, and we look forward to receiving your submissions.

## Competing interests

EJM and SS are Editors-in-Chief of *Conflict and Health*. JJO is an Editorial Board member of *Conflict and Health*.

## References

[B1] Universal Declaration of Human Rights. December 10. 1948. http://www.udhr.org/UDHR/udhr.HTM#01.

[B2] Convention on the Prevention and Punishment of the Crime of Genocide, December 9 1948. http://www.hrweb.org/legal/genocide.html.

[B3] Geneva Convention for the Amelioration of the Condition of the Wounded and Sick in Armed Forces in the Field. http://www.unhchr.ch/html/menu3/b/q_genev1.htm.

[B4] Mills E (2006). Health, Human Rights, and the Clash with Complacency. The Lancet.

[B5] Trouillier P, Olliaro P, Torreele E, Orbinski JJ, Laing R, Ford N (2002). Drug development for neglected diseases: a deficient market and a public-health policy failure. The Lancet.

[B6] Spiegel P, Le P, Ververs MT, Salama P (2007). Occurrence and overlap of natural disasters, complex emergencies and epidemics during the past decade (1995–2004). Confl Health.

[B7] Ellman T, Culbert H, Torres-Faced V (2005). Treatment of AIDS in conflict-affected settings: a failure of imagination. The Lancet.

[B8] Engelward BP, Roberts RJ (2007). Open access to research is in the public interest. PLoS Biol.

